# Supplementary figures and images for: CMTM6 is highly expressed in lung adenocarcinoma and can be used as a biomarker of a poor diagnosis
Source: PeerJ. 2023 Jan 10;11:e14668. doi: 10.7717/peerj.14668 (PMC9838204; doi:10.7717/peerj.14668)

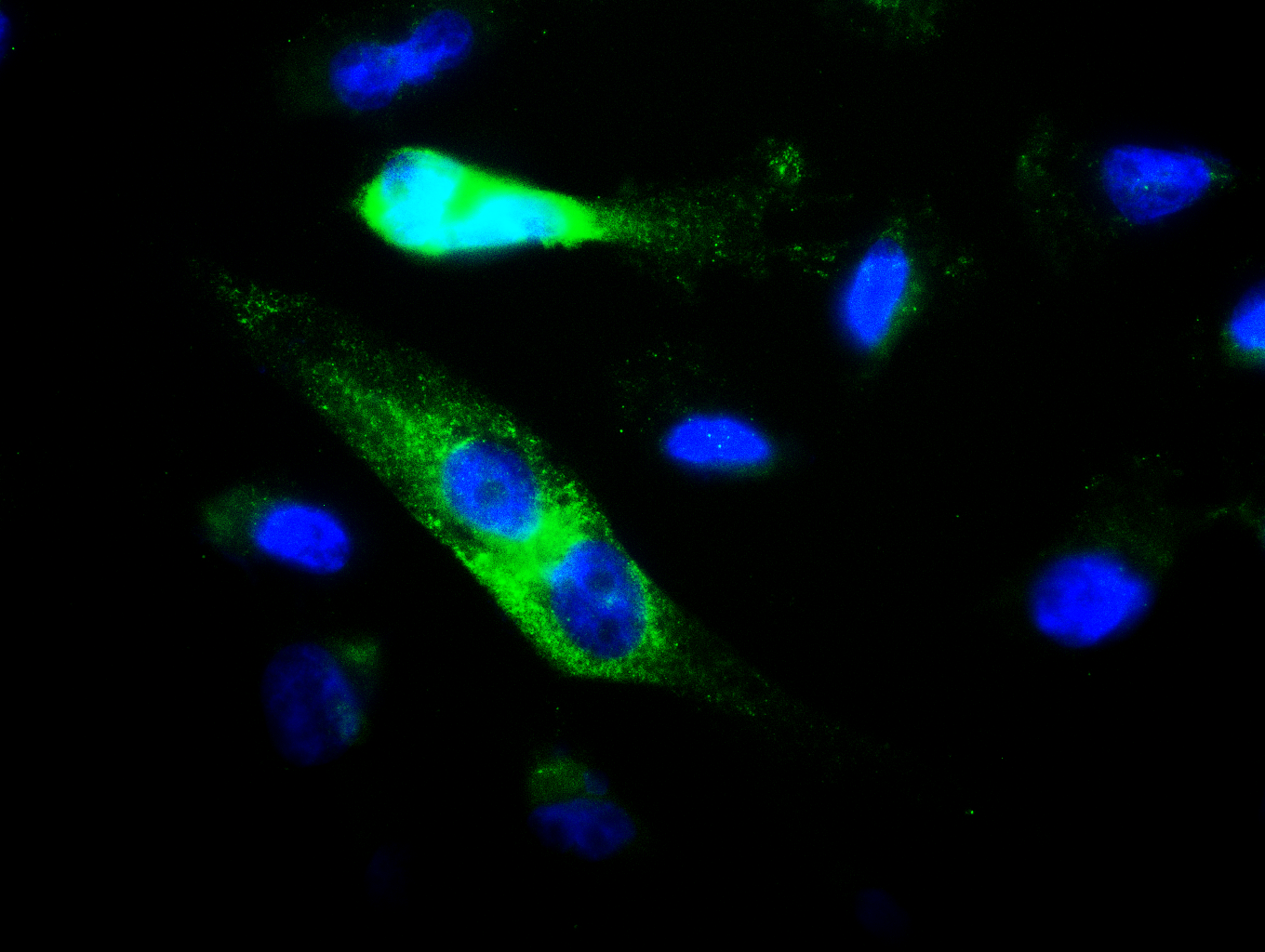

Supplement: Supplemental Information 1 [file peerj-11-14668-s001.zip › IF/A549 merge 100×.tif]

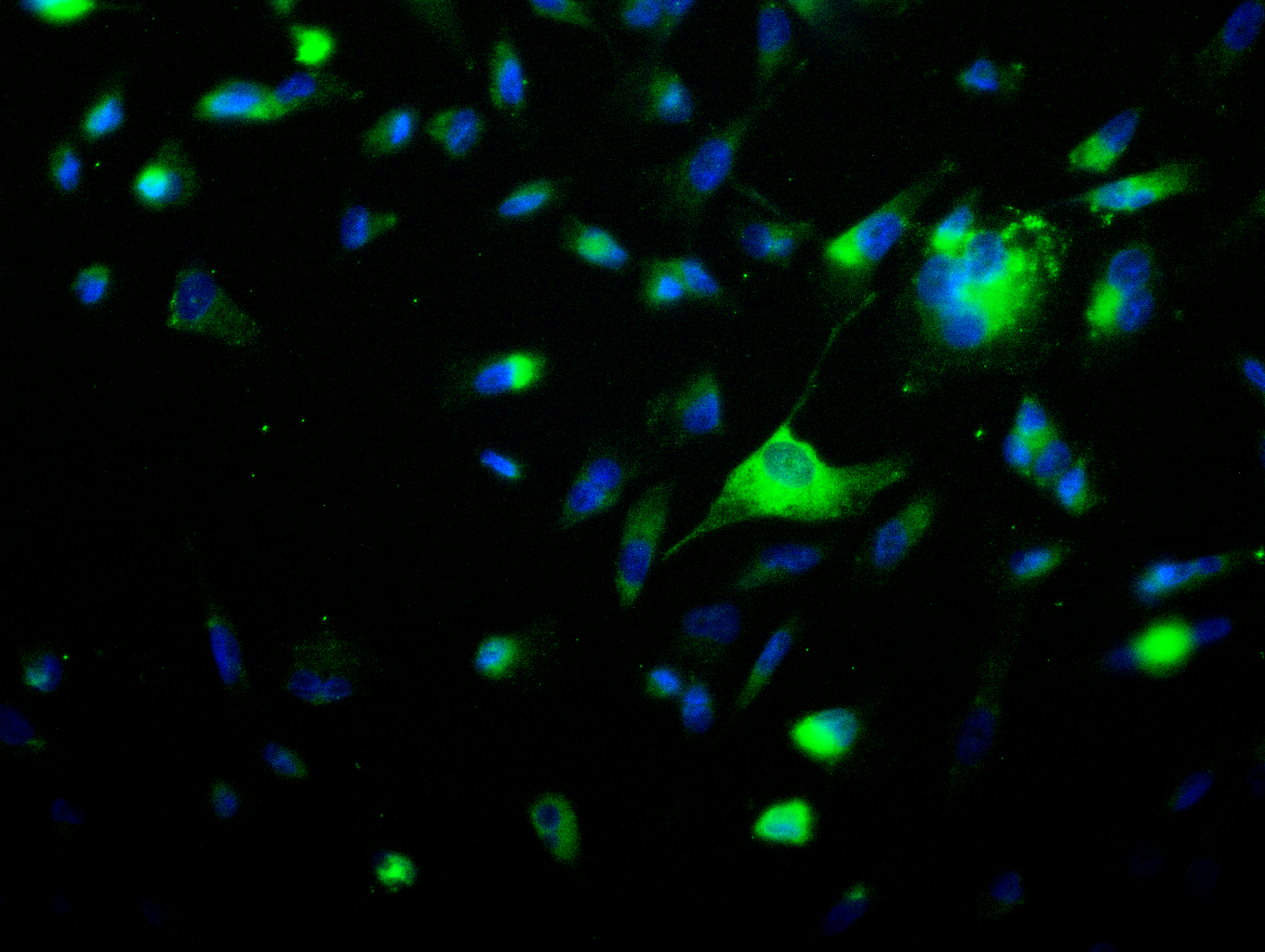

Supplement: Supplemental Information 1 [file peerj-11-14668-s001.zip › IF/A549 merge 40×.tif]

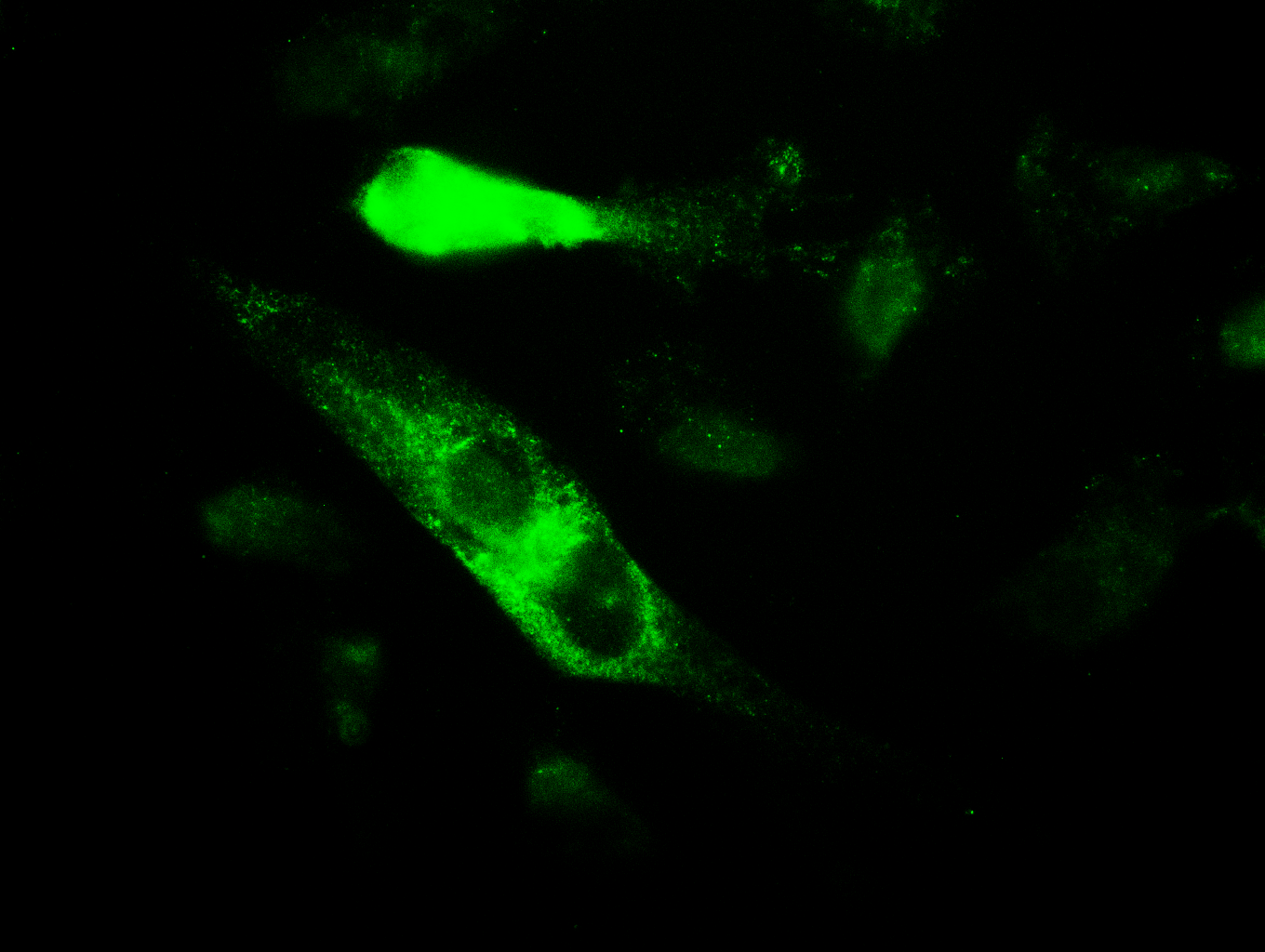

Supplement: Supplemental Information 1 [file peerj-11-14668-s001.zip › IF/A549-CMTM6 100×.tif]

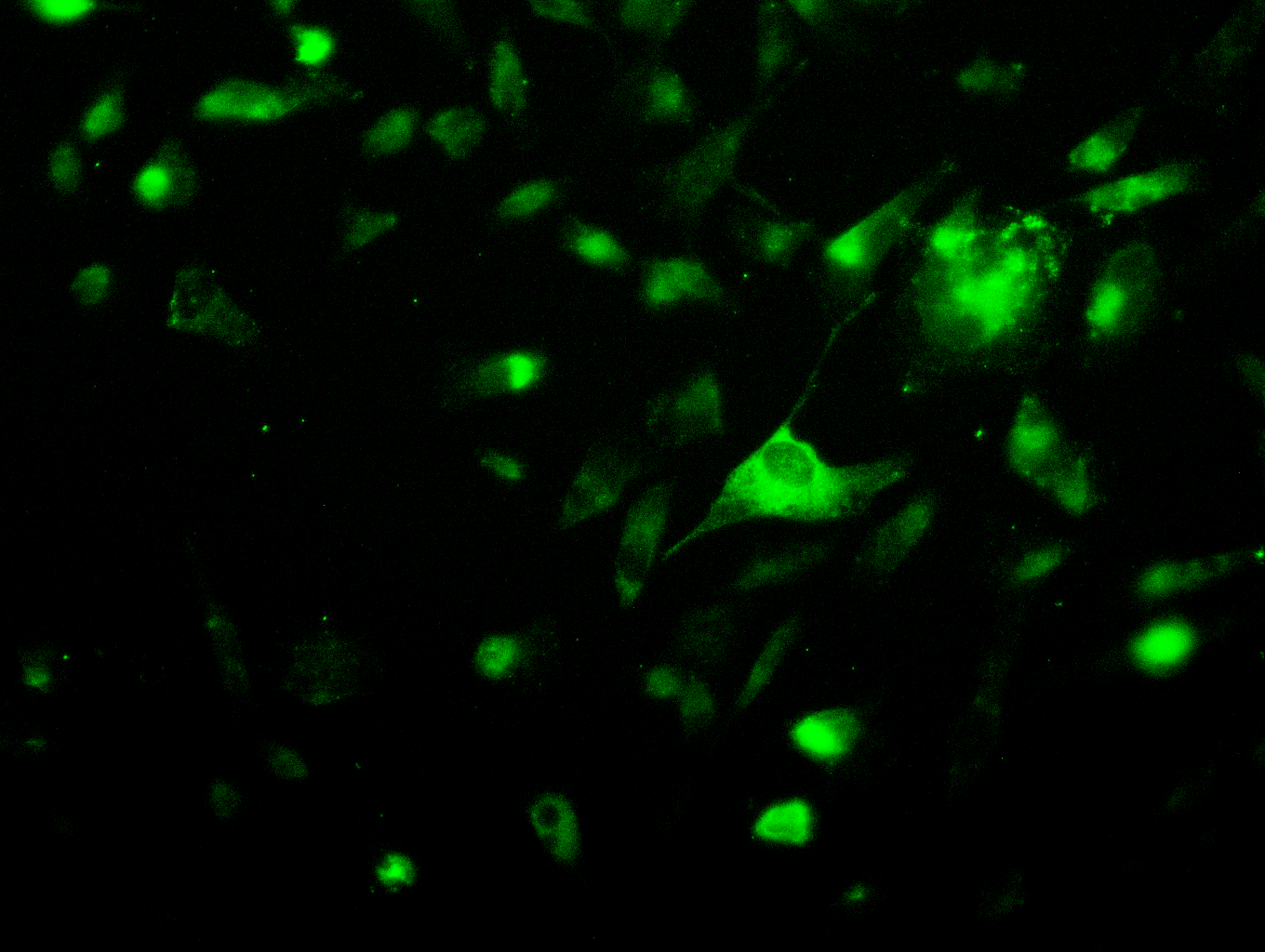

Supplement: Supplemental Information 1 [file peerj-11-14668-s001.zip › IF/A549-CMTM6 40×.tif]

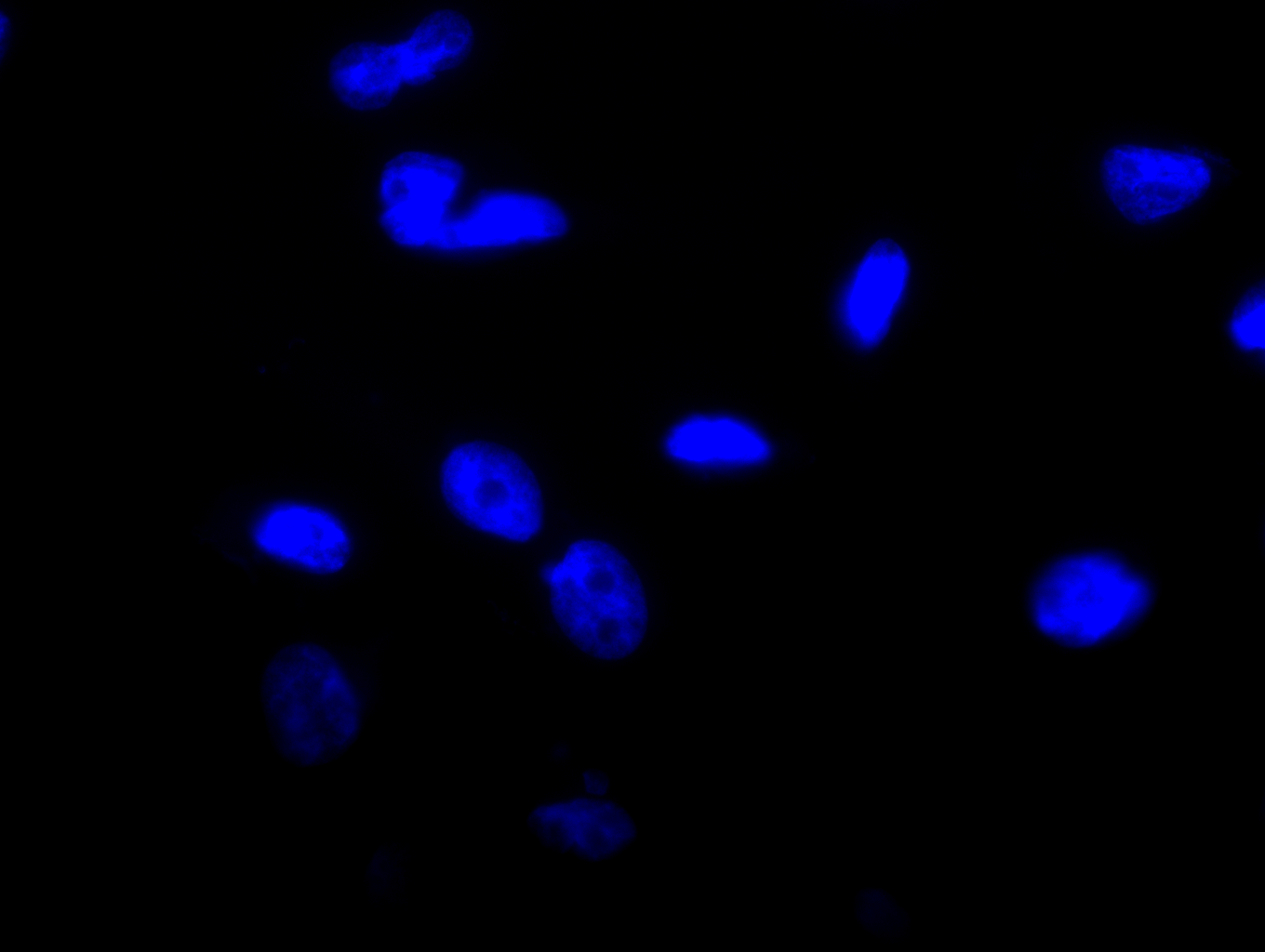

Supplement: Supplemental Information 1 [file peerj-11-14668-s001.zip › IF/A549-dapi 100×.TIF]

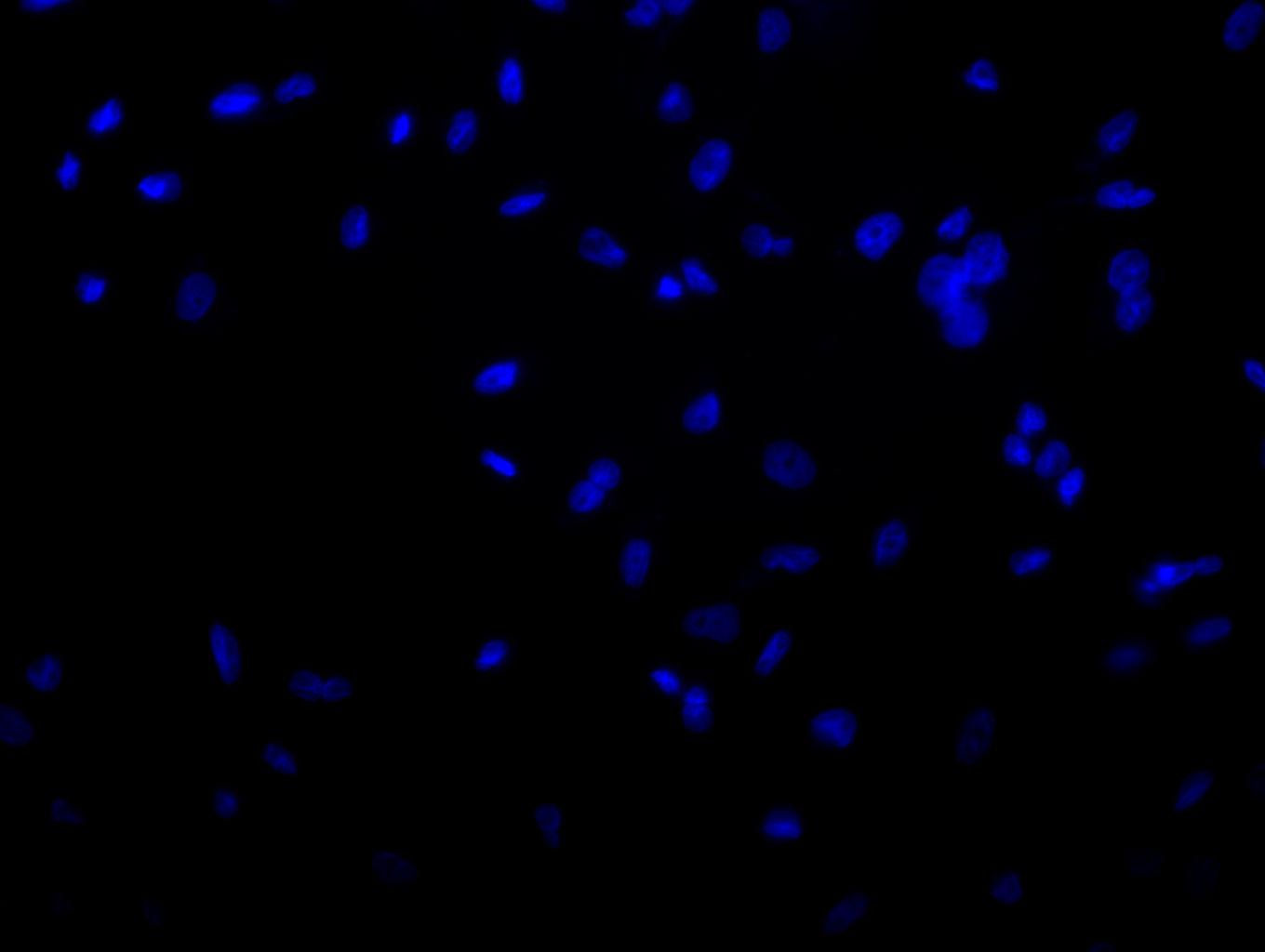

Supplement: Supplemental Information 1 [file peerj-11-14668-s001.zip › IF/A549-dapi 40×.TIF]

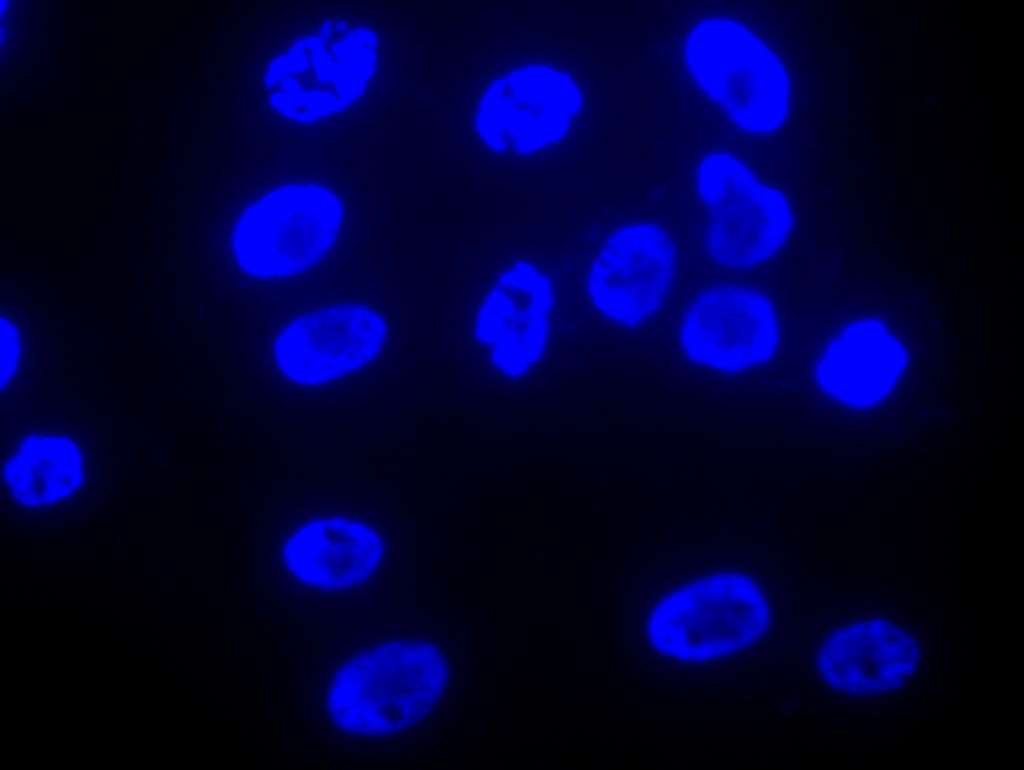

Supplement: Supplemental Information 1 [file peerj-11-14668-s001.zip › IF/Beas-2B dapi 100×.tif]

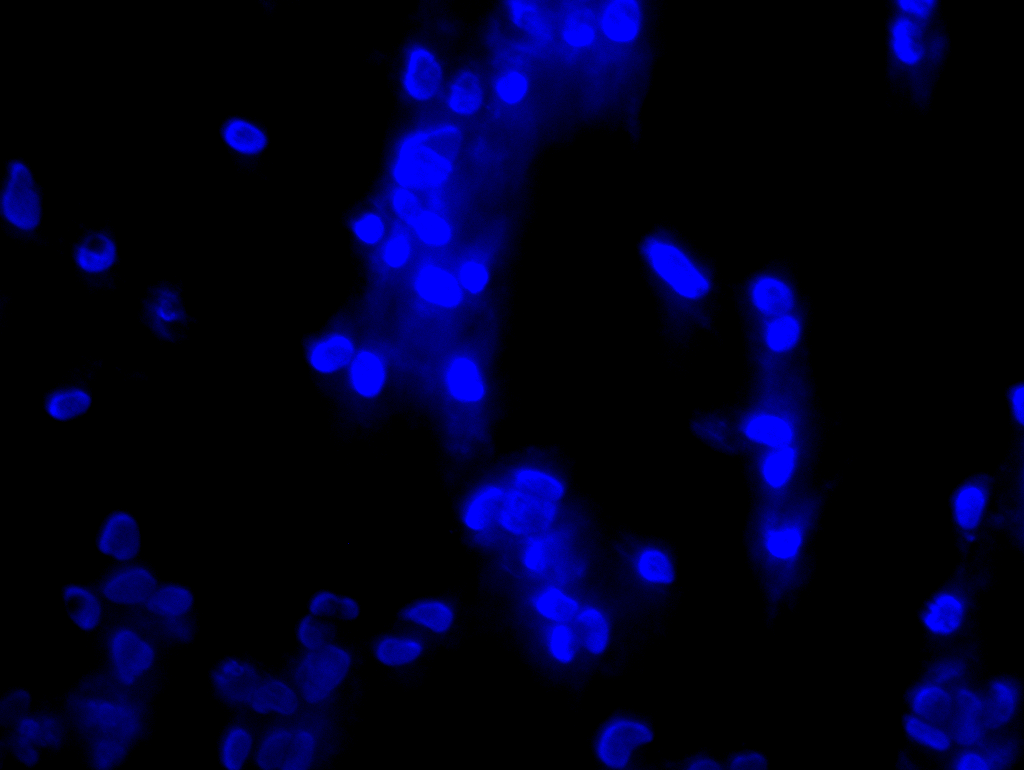

Supplement: Supplemental Information 1 [file peerj-11-14668-s001.zip › IF/Beas-2B dapi 40×.tif]

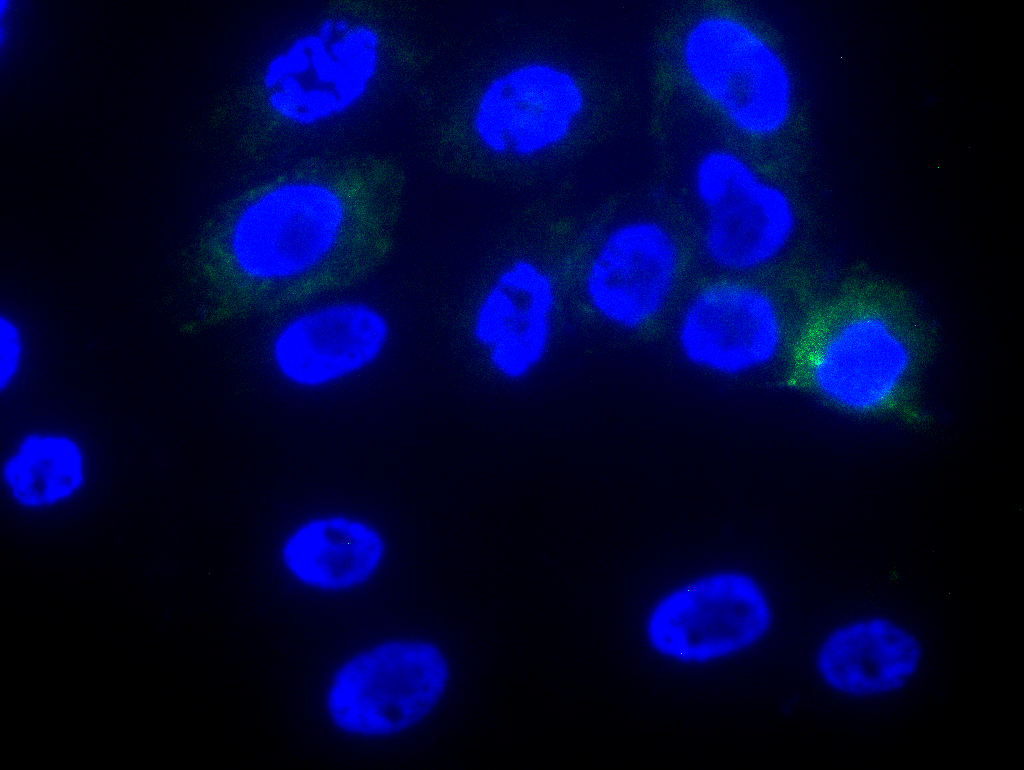

Supplement: Supplemental Information 1 [file peerj-11-14668-s001.zip › IF/Beas-2B merge 100×.tif]

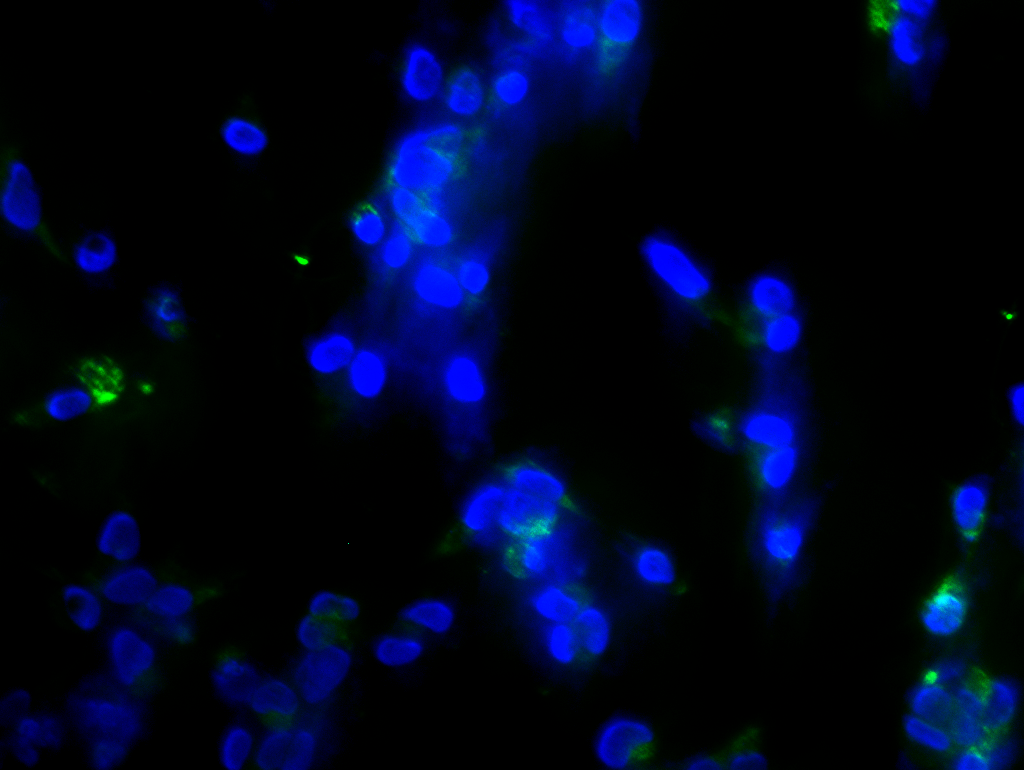

Supplement: Supplemental Information 1 [file peerj-11-14668-s001.zip › IF/Beas-2B merge 40×.tif]

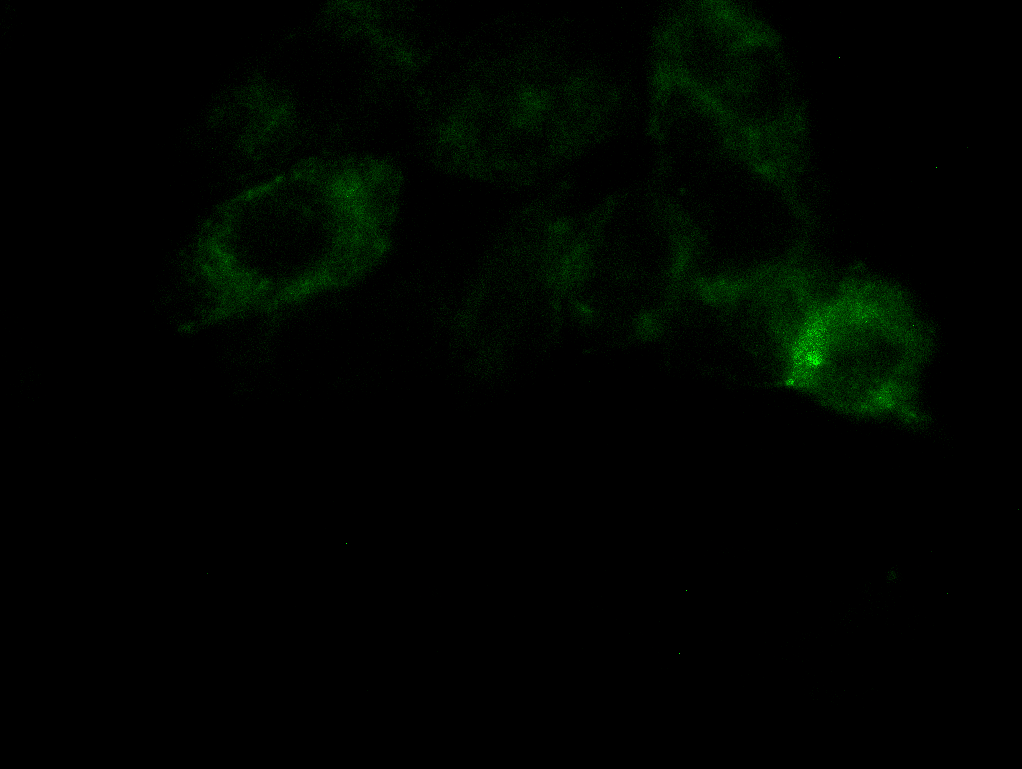

Supplement: Supplemental Information 1 [file peerj-11-14668-s001.zip › IF/Beas-2B-CMTM6 100×.tif]

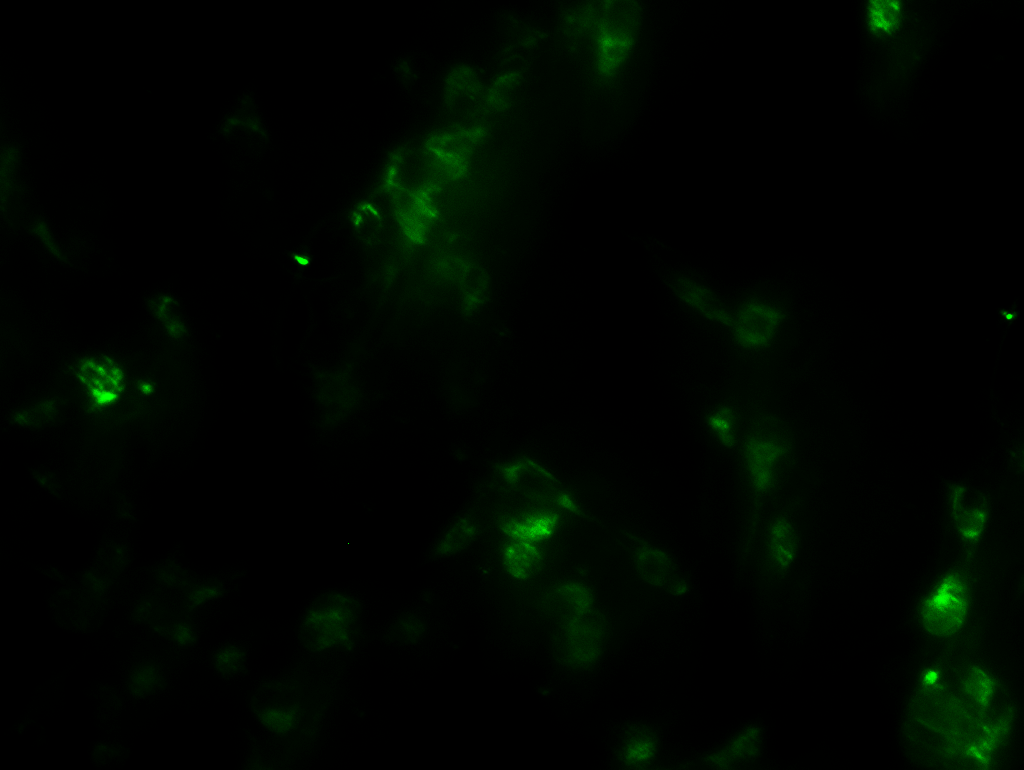

Supplement: Supplemental Information 1 [file peerj-11-14668-s001.zip › IF/Beas-2B-CMTM6 40×.tif]

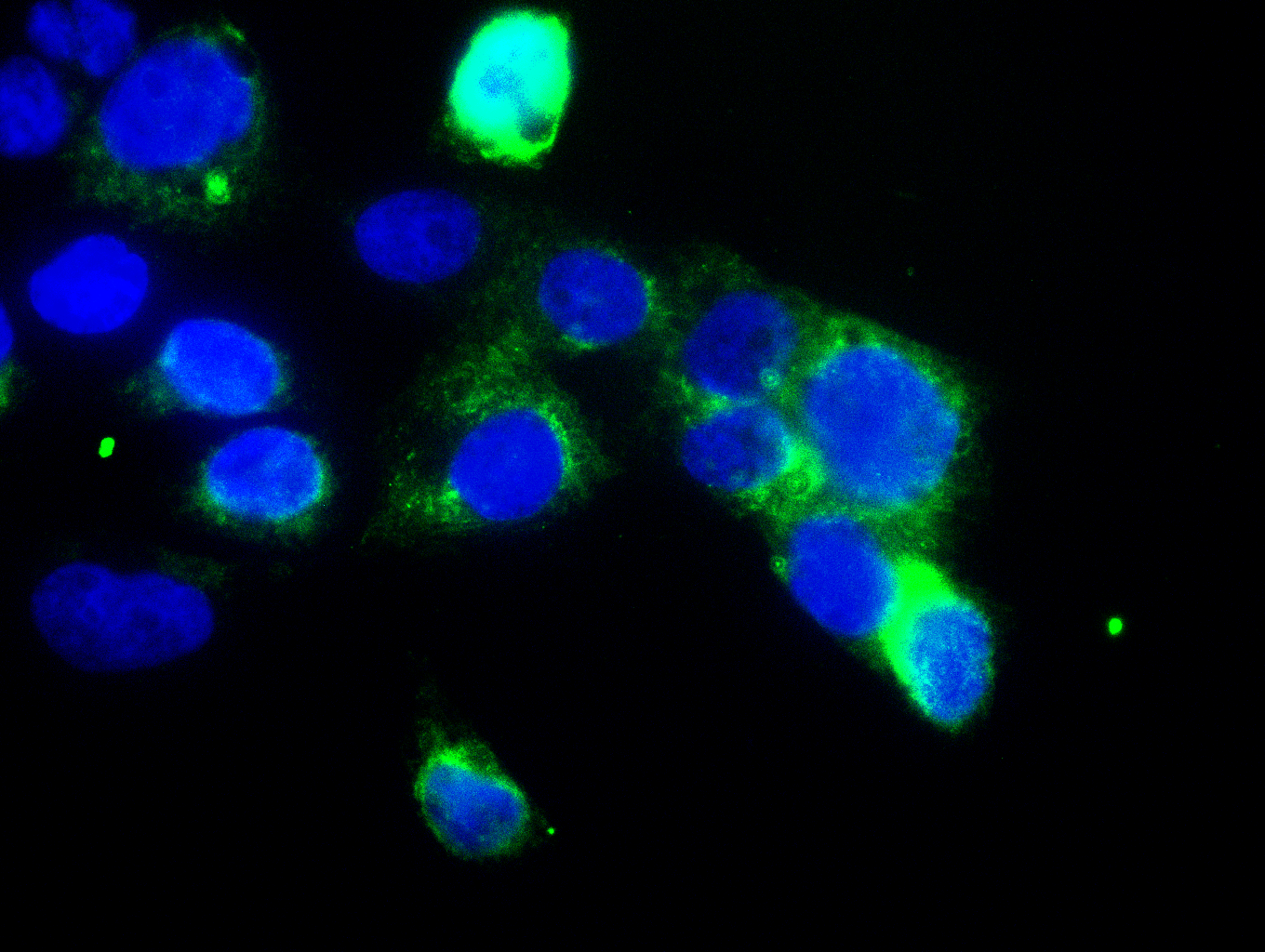

Supplement: Supplemental Information 1 [file peerj-11-14668-s001.zip › IF/H358 merge 100×.tif]

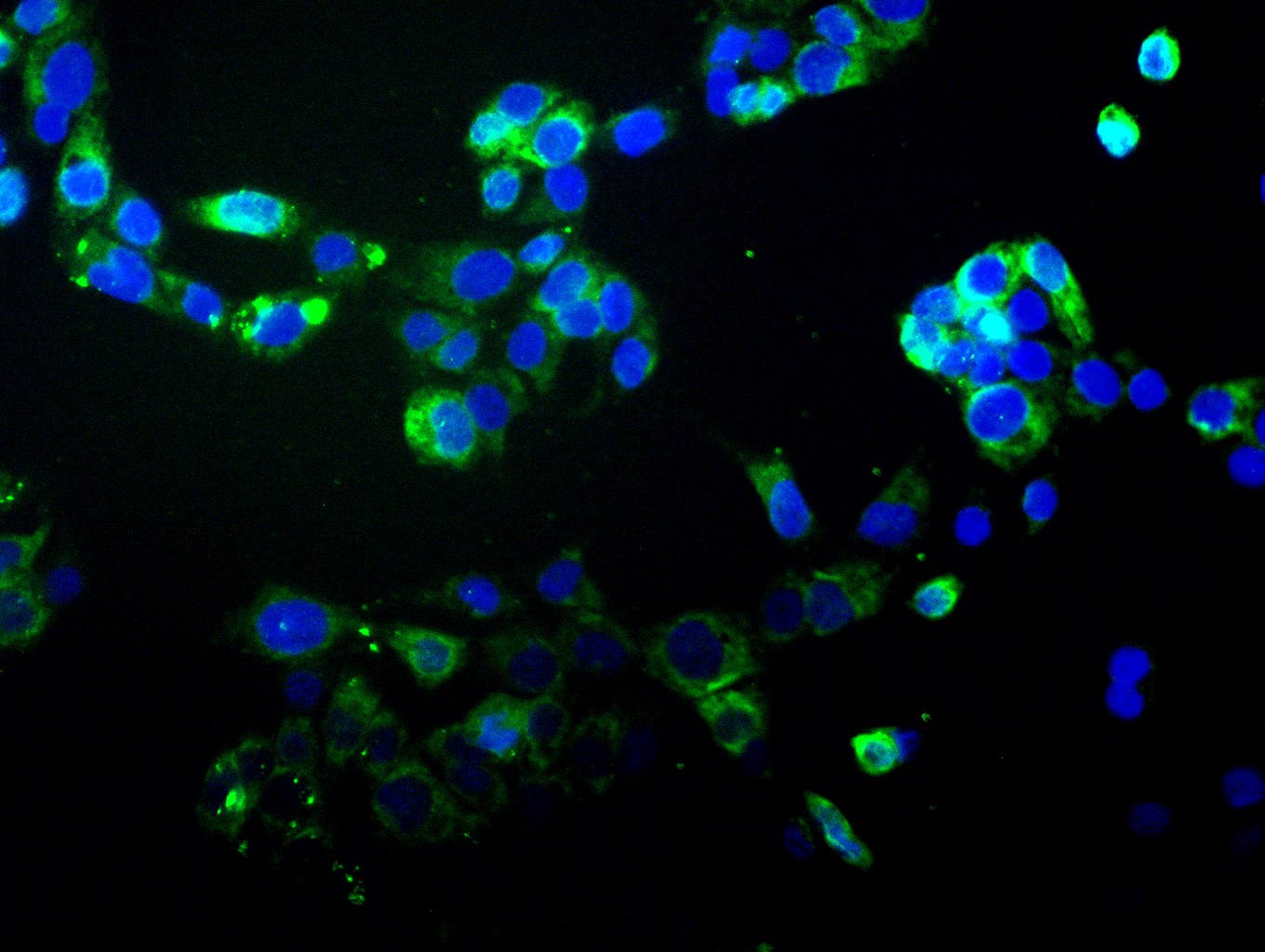

Supplement: Supplemental Information 1 [file peerj-11-14668-s001.zip › IF/H358 merge 40×.tif]

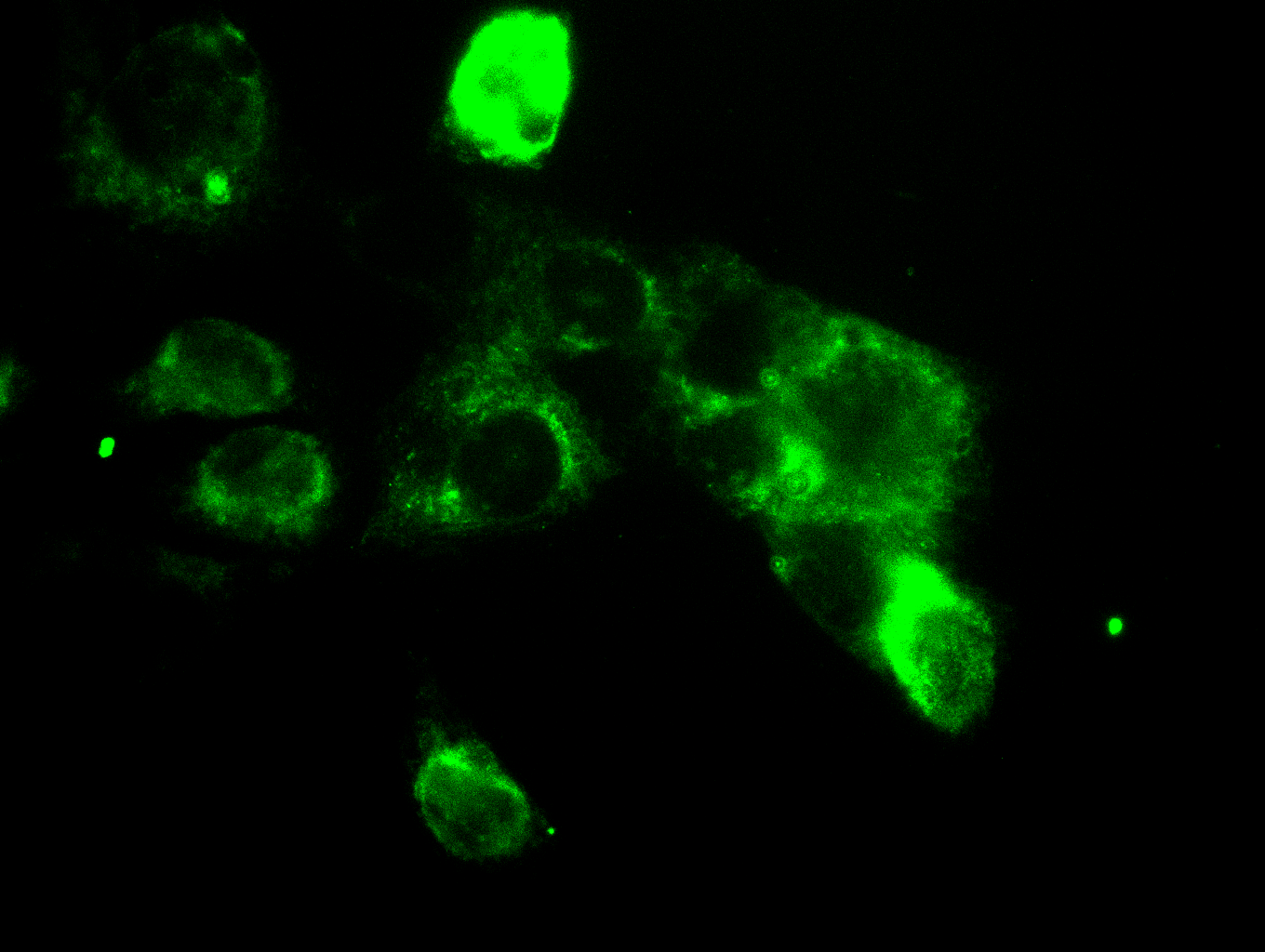

Supplement: Supplemental Information 1 [file peerj-11-14668-s001.zip › IF/H358-CMTM6 100×.TIF]

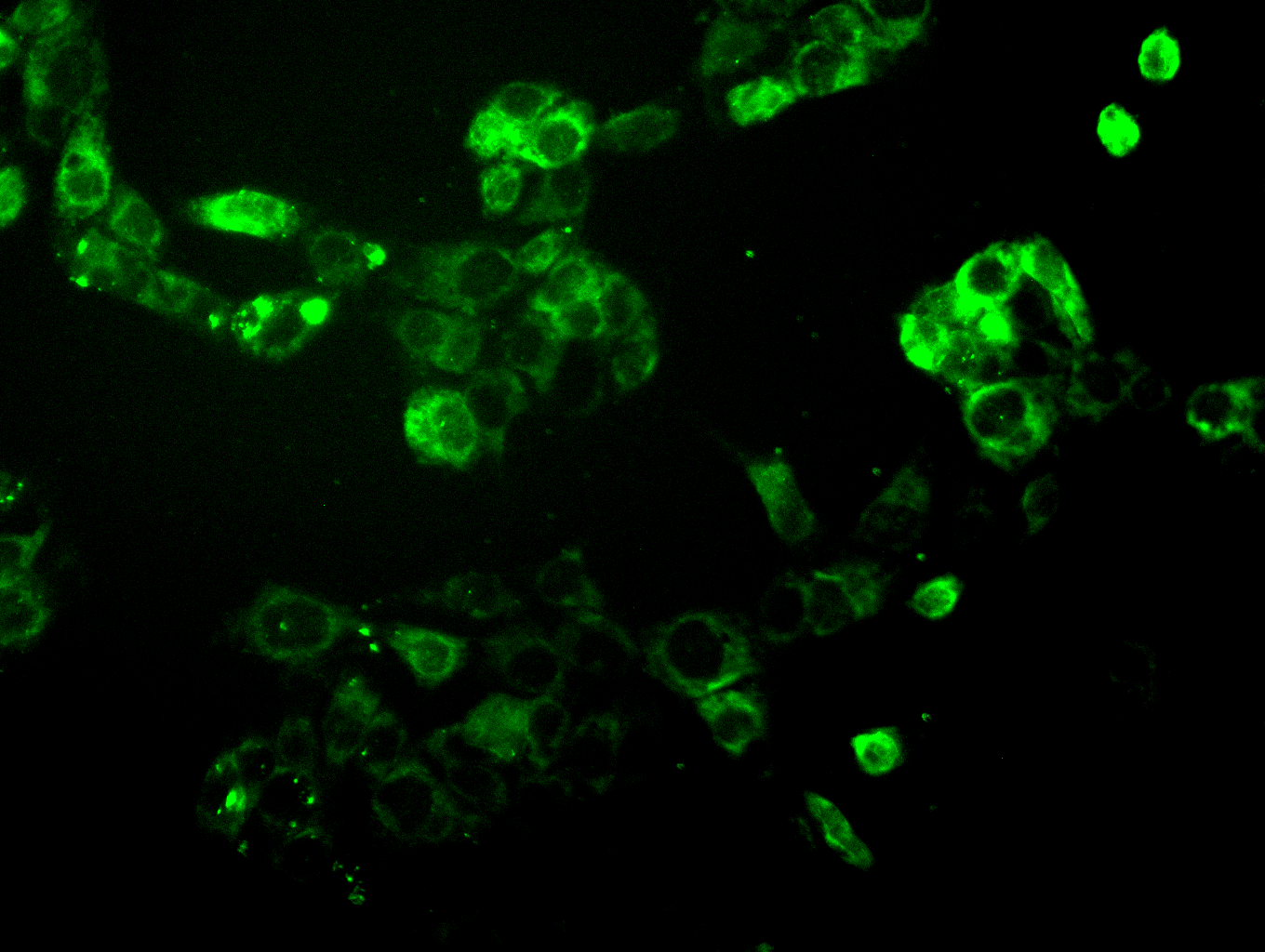

Supplement: Supplemental Information 1 [file peerj-11-14668-s001.zip › IF/H358-CMTM6 40×.TIF]

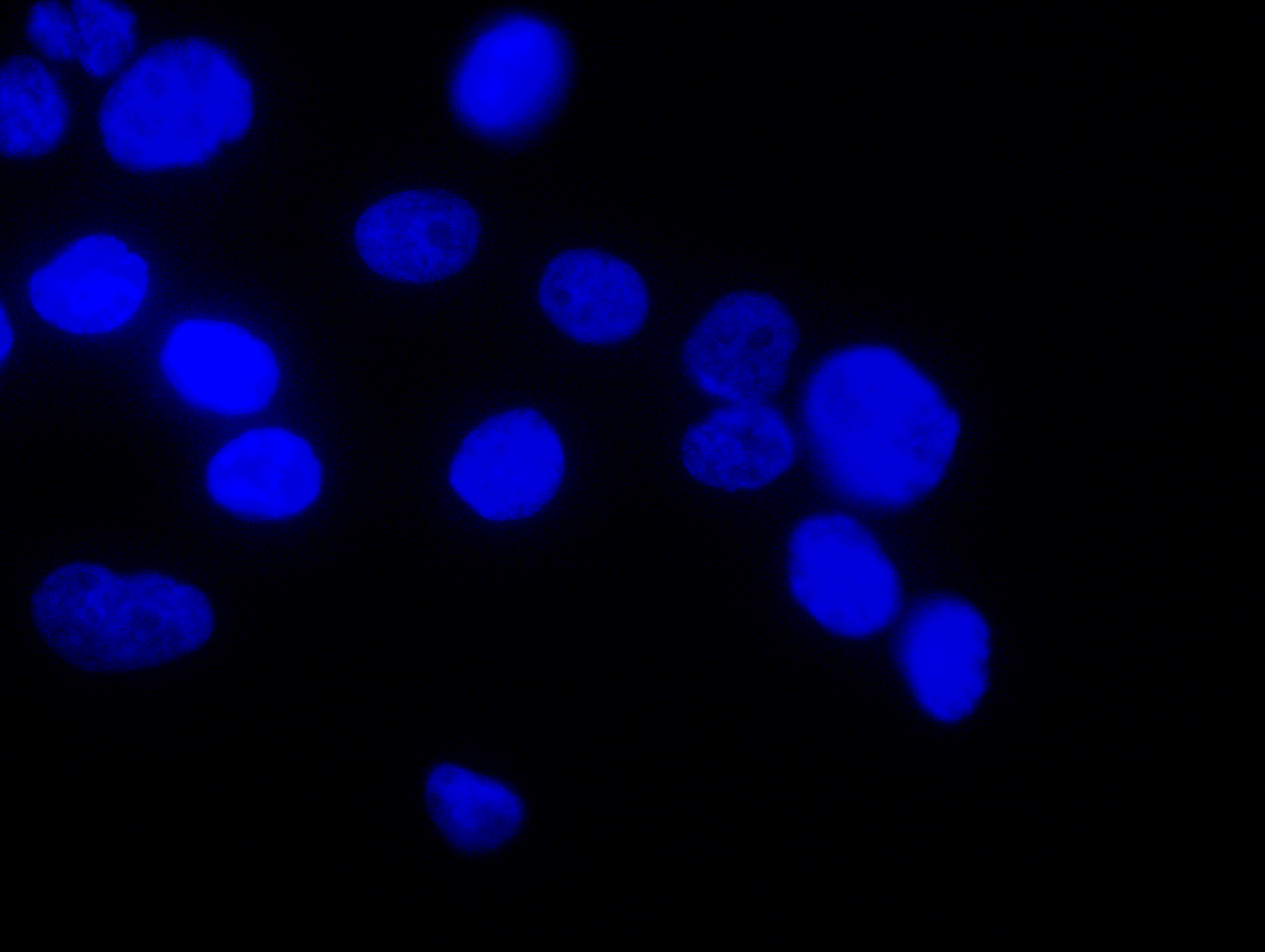

Supplement: Supplemental Information 1 [file peerj-11-14668-s001.zip › IF/H358-dapi 100×.TIF]

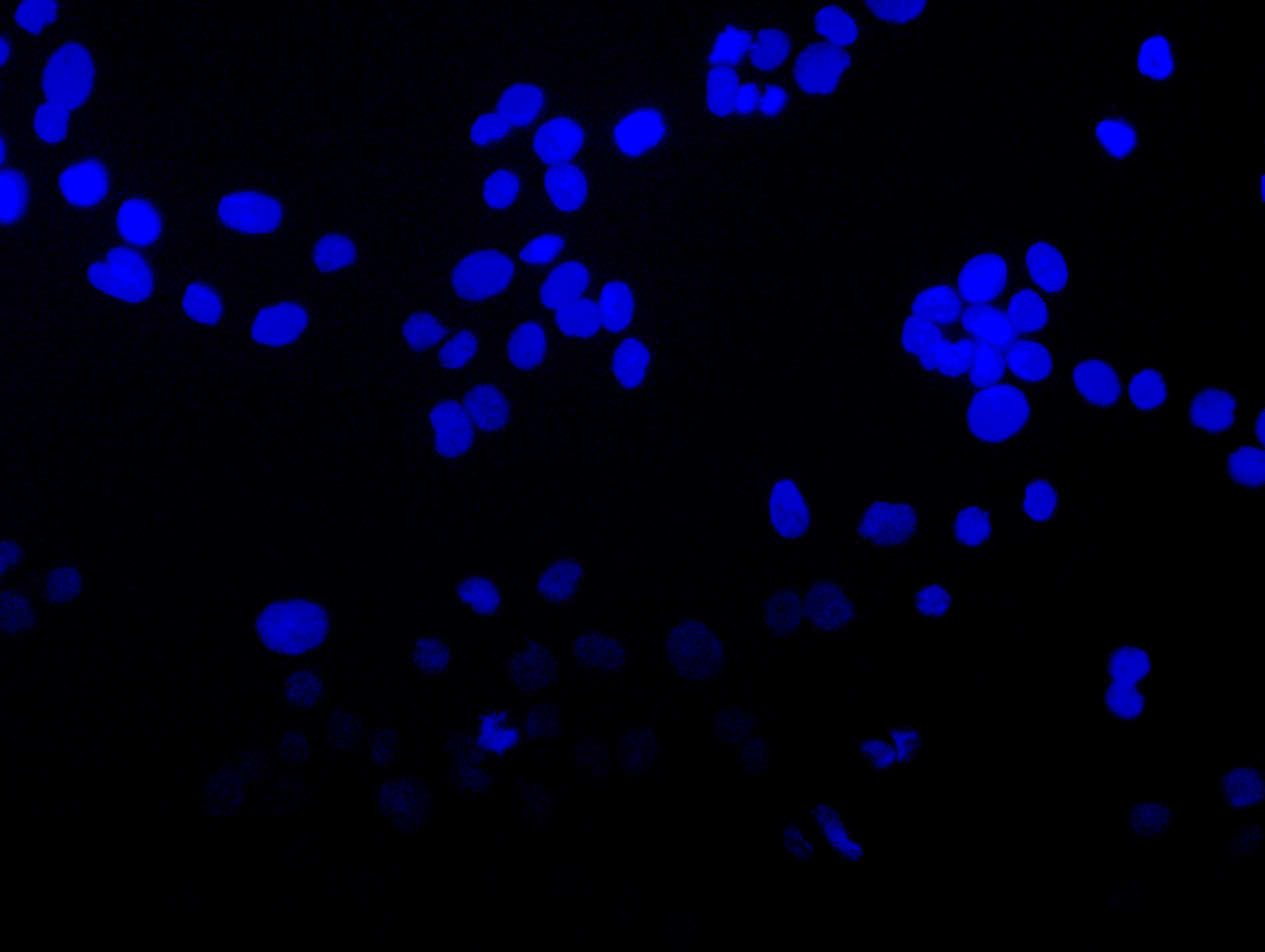

Supplement: Supplemental Information 1 [file peerj-11-14668-s001.zip › IF/H358-dapi 40×.TIF]

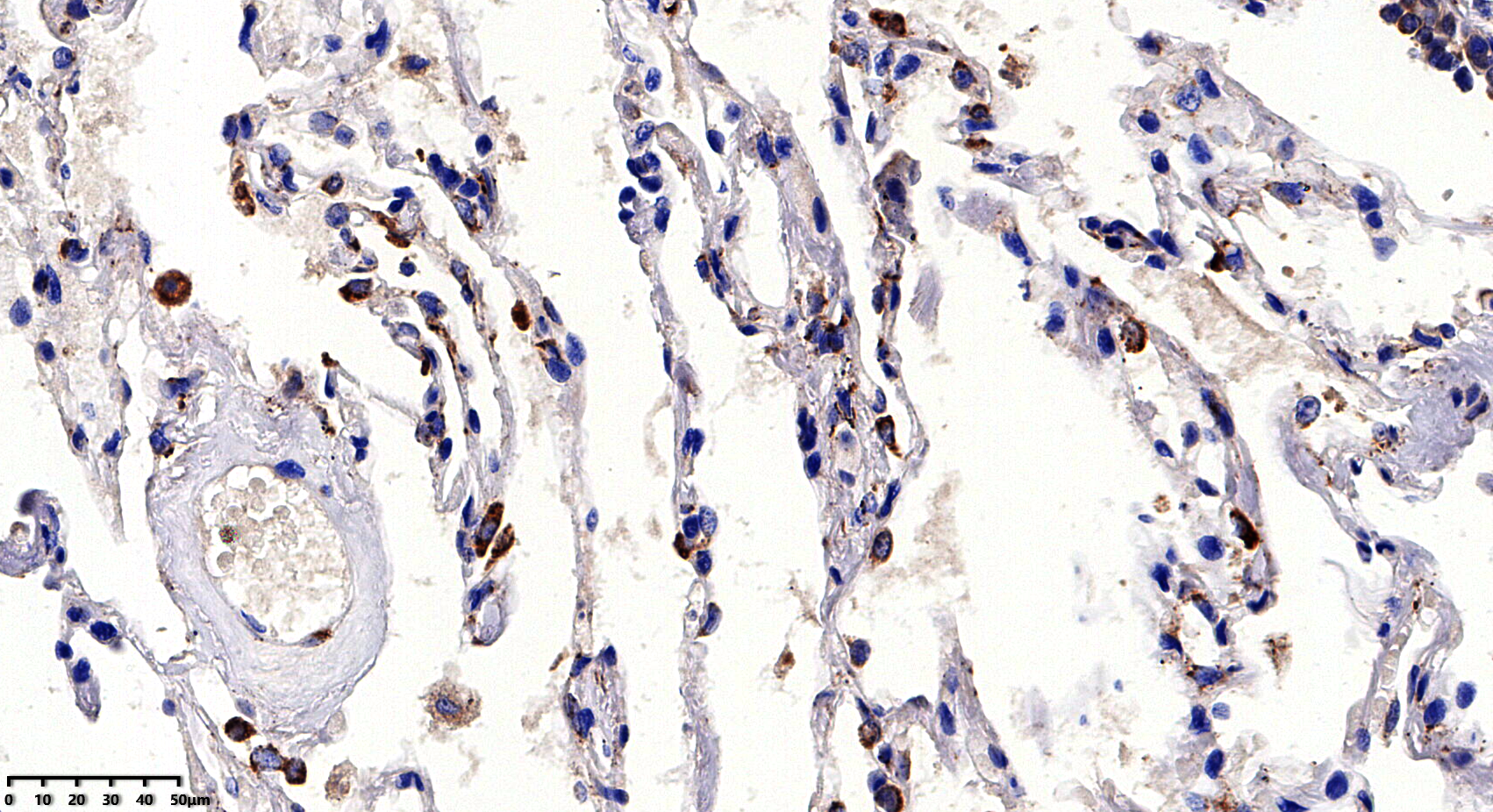

Supplement: Supplemental Information 1 [file peerj-11-14668-s001.zip › IHC/Paracarinoma low CMTM6 expression-1.tiff]

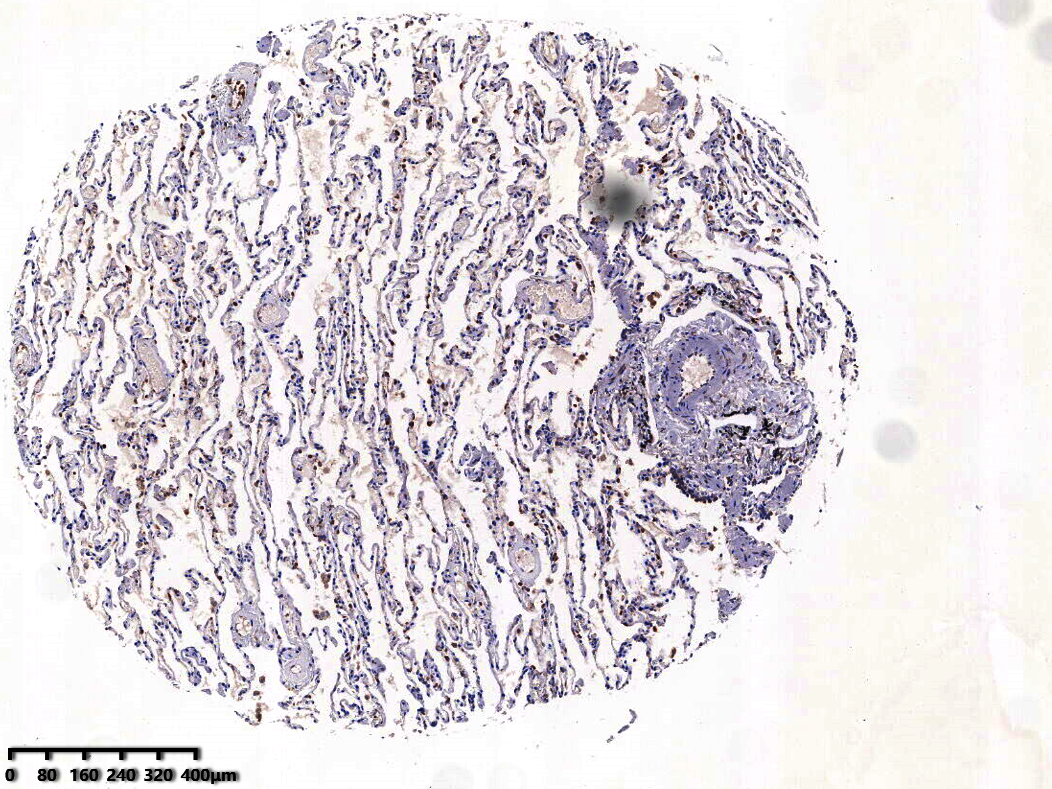

Supplement: Supplemental Information 1 [file peerj-11-14668-s001.zip › IHC/Paracarinoma low CMTM6 expression.tiff]

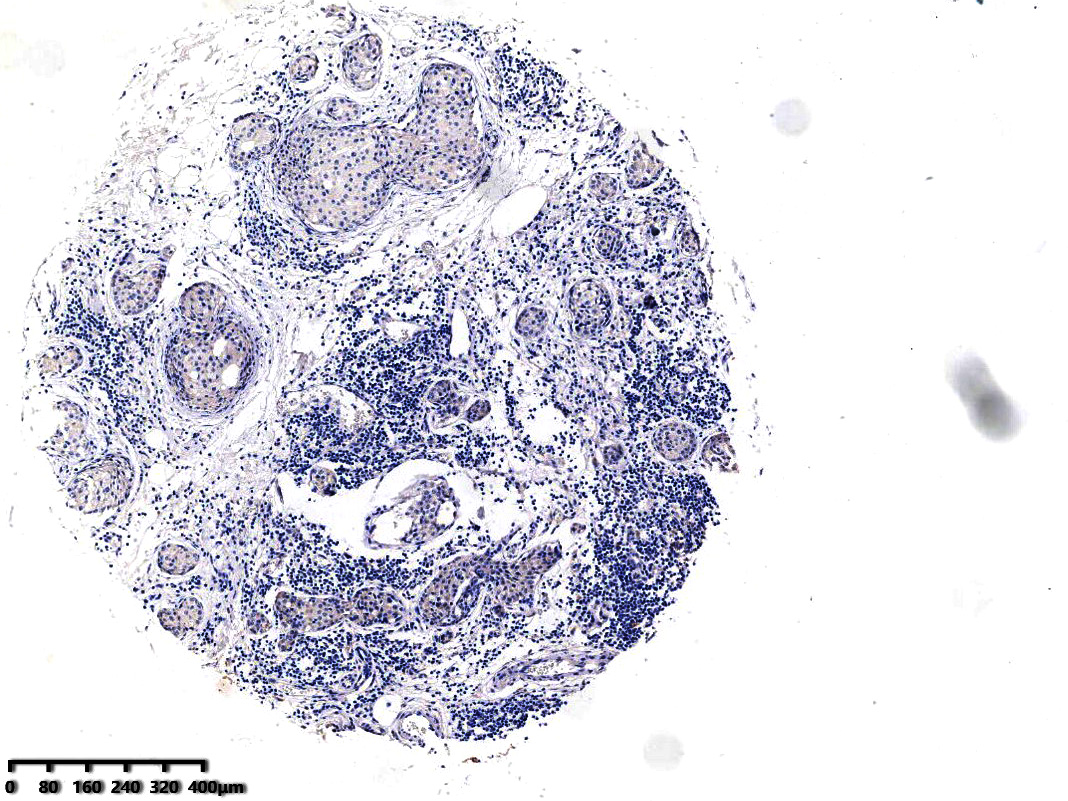

Supplement: Supplemental Information 1 [file peerj-11-14668-s001.zip › IHC/Tumor #1 low CMTM6 expression.jpg]

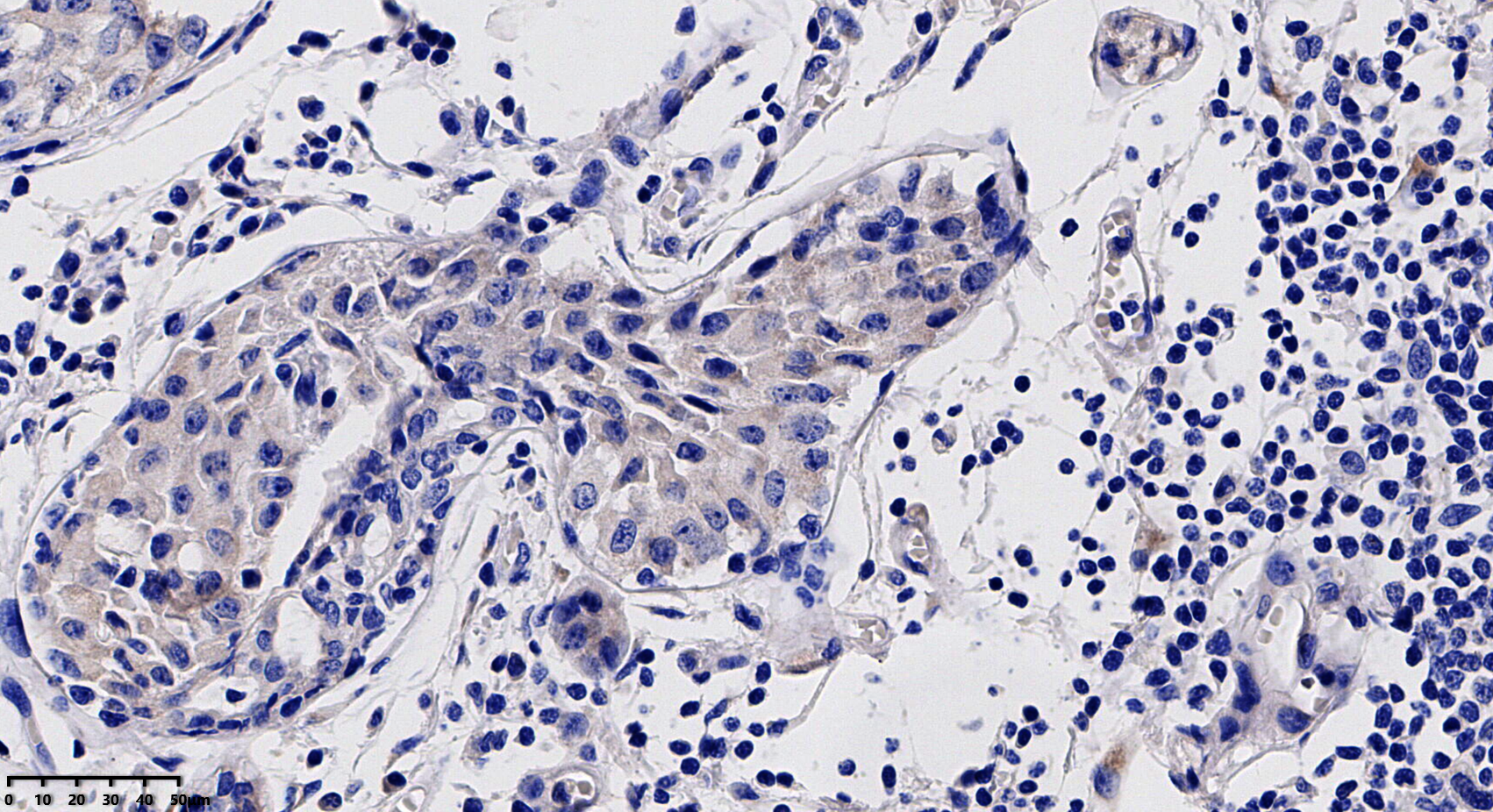

Supplement: Supplemental Information 1 [file peerj-11-14668-s001.zip › IHC/Tumor #1-1low CMTM6 expression.tiff]

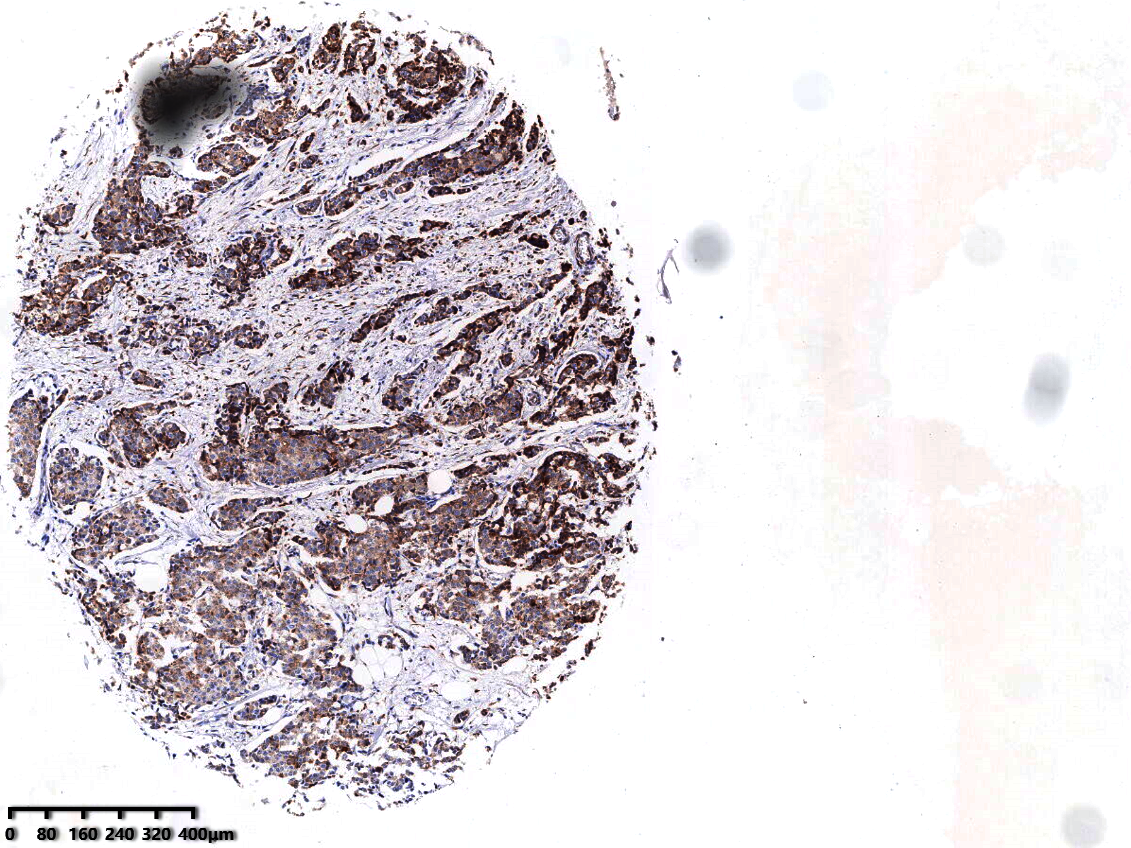

Supplement: Supplemental Information 1 [file peerj-11-14668-s001.zip › IHC/Tumor #2 high CMTM6 expression.tiff]

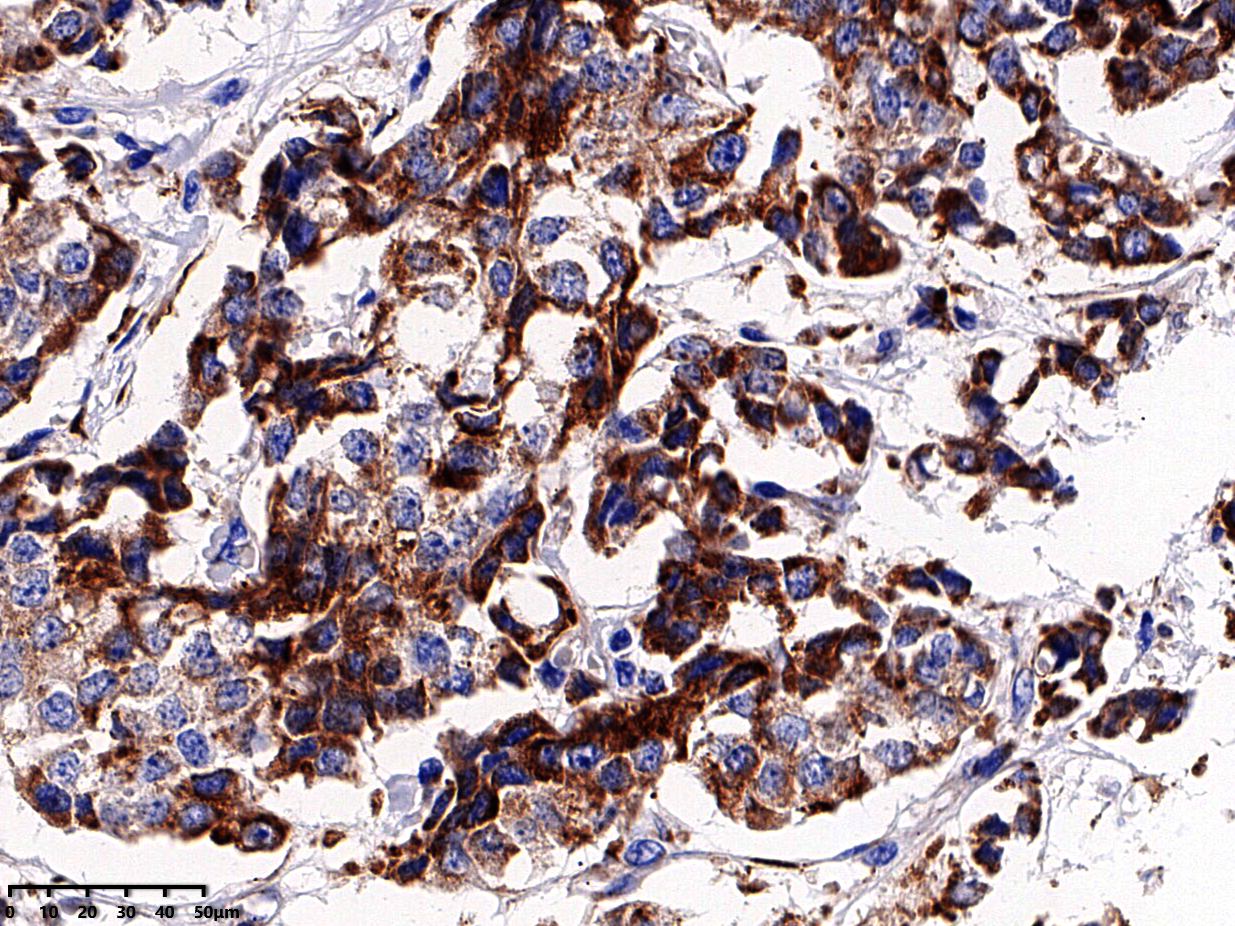

Supplement: Supplemental Information 1 [file peerj-11-14668-s001.zip › IHC/Tumor#2-1 high CMTM6 expression.tiff]

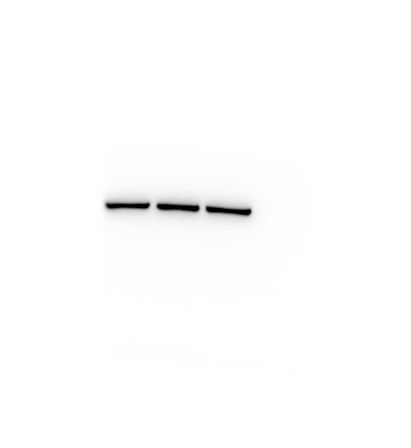

Supplement: Supplemental Information 1 [file peerj-11-14668-s001.zip › WB/ACTIN.JPG]

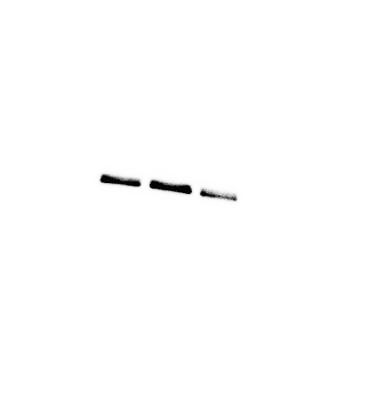

Supplement: Supplemental Information 1 [file peerj-11-14668-s001.zip › WB/CMTM6.JPG]
